# *In-Vivo* Induced CAR-T Cell for the Potential Breakthrough to Overcome the Barriers of Current CAR-T Cell Therapy

**DOI:** 10.3389/fonc.2022.809754

**Published:** 2022-02-10

**Authors:** Tianqing Xin, Li Cheng, Chuchao Zhou, Yimeng Zhao, Zhenhua Hu, Xiaoyan Wu

**Affiliations:** ^1^Department of Pediatrics, Tongji Medical College, Union Hospital, Huazhong University of Science and Technology, Wuhan, China; ^2^Department of Health and Nursing, Nanfang College of Sun Yat-sen University, Guangzhou, China

**Keywords:** CAR-T cells, barriers, *in-situ* editing, gene-editing tool, nano-delivery

## Abstract

Chimeric antigen receptor T cell (CAR-T cell) therapy has shown impressive success in the treatment of hematological malignancies, but the systemic toxicity and complex manufacturing process of current autologous CAR-T cell therapy hinder its broader applications. Universal CAR-T cells have been developed to simplify the production process through isolation and editing of allogeneic T cells from healthy persons, but the allogeneic CAR-T cells have recently encountered safety concerns, and clinical trials have been halted by the FDA. Thus, there is an urgent need to seek new ways to overcome the barriers of current CAR-T cell therapy. *In-vivo* CAR-T cells induced by nanocarriers loaded with CAR-genes and gene-editing tools have shown efficiency for regressing leukemia and reducing systemic toxicity in a mouse model. The *in-situ* programming of autologous T-cells avoids the safety concerns of allogeneic T cells, and the manufacture of nanocarriers can be easily standardized. Therefore, the *in-vivo* induced CAR-T cells can potentially overcome the abovementioned limitations of current CAR-T cell therapy. Here, we provide a review on CAR structures, gene-editing tools, and gene delivery techniques applied in immunotherapy to help design and develop new *in-vivo* induced CAR-T cells.

## Introduction

Chimeric antigen receptor T cell (CAR-T cell) therapy is a new cell immunotherapy technique that incorporates synthetic receptors into T cells that recognize and kill tumor cells with a cognate targeting ligand (1, 2). CAR-T cell therapy has demonstrated unprecedented response rates in patients with B cell lymphoma since the first approval of CD19-targeted CAR-T cells in the USA (1, 3–5). However, along with the remarkable achievements of CAR-T cell therapy, many systemic toxicities, such as cytokine release syndrome (CRS) and neurotoxicity, have also been frequently reported (2, 6–8). Additionally, the complex manufacturing process of CAR-T cells limits the broader applications of this therapeutic method as a standard clinical treatment (2, 9–11). Therefore, there is an exigent need to develop a new paradigm of CAR-T cells to overcome these barriers and allow this therapeutic method to benefit more patients. To simplify the complex manufacturing process of CAR-T cells, universal allogeneic CAR-T cells from healthy persons have been tested in clinical trials (12–15). Universal CAR-T cells can be off-the-shelf and then infused into patients like usual medicines, without needing to wait for the isolation of autologous T cells from patients (12, 16); however, last year’s death case during the clinical trial of UCARTCS1A from Cellectis raised safety concerns about allogeneic CAR-T cells. The FDA also recently halted all clinical trials on universal CAR-T cells from Allogene due to safety concerns (17). Thus, we need new strategies to overcome the associated toxicity and simplify the manufacturing process of current CAR-T cell therapy. *In-vivo* CAR-T cells induced by nanocarriers loaded with CAR genes and gene-editing tools have shown promising effects for regressing leukemia (18–20). The *in-situ* programming of autologous CAR-T cells can enhance the targeted killing of tumor cells and reduce systemic toxicity such as CRS and neurotoxicity. Additionally, the nanocarriers can be easily manufactured in a standardized method (21) *In-vivo* induced CAR-T cells provide a potential solution to overcome the barriers of current CAR-T cell therapy. Thus, here, we review CAR structure design, gene-editing tools, and gene delivery systems and the future trend of immune cell therapy.

## CAR Structure and Evolution

**Figure 1:**
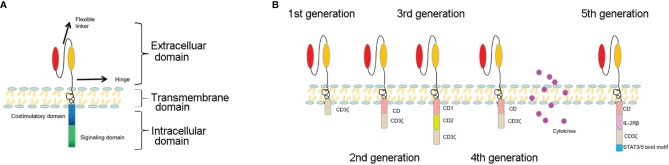
**(A)** The basic structure of a CAR: extracellular domain, transmembrane domain, and intracellular domain. **(B)** The development of the five generations of CARs. The first generation only contained the CD3ζ chain functional energy domain9; the second generation contained CD3ζ+ a costimulatory molecular domain (CD28, 4-1BB, etc.); the third generation contained CD3ζ and two costimulatory molecular domains; the fourth generation included suicide gene editing, immune factor modification, and other integrated and refined regulatory tools; the fifth generation included simultaneous activation of TCR, costimulatory domain, and cytokine triple signaling.

The structure of the chimeric antigen receptor (CAR) has a modular design consisting of an antigen-binding domain, a hinge, a transmembrane domain, and an intracellular signaling domain (**Figure 1A**). The antigen-binding domain is usually a single-chain variable fragment (scFv) molecule derived from a monoclonal antibody that can bind to antigens on the surface of malignant cancer cells (4, 22–24). The transmembrane domain is responsible for anchoring the CAR onto the T cell membrane. The intracellular signaling domain generally contains a T cell activation domain derived from the CD3ζ chain of the T cell receptor as well as co-stimulatory domains often comprised of an immunoreceptor tyrosine-based activation motif containing regions of CD28 or 4-1BB (also known as CD137 and TNFRSF9) (25–29). Variations in each component of the CAR structure enable fine-tuning of the functionality and antitumor activity of the resultant CAR-T cell product. Various CAR structures have been designed to improve the safety and efficacy of CAR-T cell therapy. Once the designed CAR genes are integrated into T cells, the scFv on the surface of T cells specifically recognizes tumor-associated antigens and binds CAR-T cells with tumor cells. After that, the intracellular signal domains of CAR-T cells are activated and cause CAR-T cells to proliferate and secrete cytokines that kill tumor cells (30–32).

There have been five generations of CAR structures since the first clinical application of CAR-T cells by Carl June at the University of Pennsylvania and hematologist David Porter at the Children’s Hospital in Philadelphia in 2011 (33–35). The first-generation CAR contained an intracellular stimulation region and an extracellular scFv. This generation of CAR-T cells could not continuously proliferate due to the lack of costimulatory molecules (**Figure 1B**) (34). The second-generation CAR added a costimulatory molecule, such as CD28, or 4-1BB (CD137) to enhance the proliferation and reduce the toxicity of CAR-T cells (36). Yescarta™ (Tisagenlecleucel) and Kymriah™ (axicabtagene ciloleucel) are second-generation CAR-T cells that contain CD28 and 4-1BB, respectively (36). The third-generation CAR includes two costimulatory molecules, such as CD27, CD28, tumor necrosis factor superfamily 4 (OX40, also known as CD134), CD137 (4-1BB), or CD244 (37, 38). The fourth-generation CAR is called TRUCKs (T cells redirected for antigen-unrestricted cytokine-initiated killing), which combines the direct antitumor capacities of CAR-T cells with the immune modulating function of the delivered cytokine (34, 39). TRUCKs have entered early-phase clinical trials using a panel of cytokines, including IL-7, IL-12, IL-15, IL-18, IL-23, and their combinations. The fifth generation integrates an additional membrane receptor that controls the activation of CAR-T cells in an antigen-dependent manner (38, 40).

In addition to adding new functional molecules into the CAR structure, many studies have chosen alternative tumor-targeted sites for new CAR structures. CD30 shows very strong expression on malignant cells in Hodgkin’s lymphoma, rather than on healthy lymphocytes and hematopoietic stem/progenitor cells (HSPCs). CD30 CAR-T cell therapy has shown superior results in the treatment of CD30^+^ malignant tumors, while healthy activated lymphocytes and HSPC were unaffected (41). CD20 is a 33–37-kDa non-glycosylated transmembrane phosphoprotein that helps develop and differentiate B cells (42). CD20 is highly expressed in late pre-B cells and mature B cells, but it is not expressed on the surface of HSPCs (43). CD20 CAR T-cell therapy which has shown promise in the treatment of B-cell non-Hodgkin lymphoma is now being considered for patients with relapsed or refractory CD20-positive chronic lymphocytic leukemia. Lym-1 targets the conformational epitopes of human leukocyte antigen D-associated antigens (HLA-DRs) on the surface of human B-cell lymphoma. The binding affinity of Lym-1 with malignant B cells is higher than that of normal B cells (44). Lym-1 CAR-T cells have exhibited potent antitumor effects against B-cell lymphoma. Some alternative targeting sites combine with CD19 to form dual-target CAR T cells. For example, CD37 combined with CD19 was incorporated into one CAR to generate a dual-specific CAR T cell capable of recognizing CD19 and CD37 alone or together (45). CD79b is also a complementary targeting site for CD19. CD19 and CD79 dual-specific CAR-T cells prevented the escape of B-cell lymphoma from a single CD19 CAR-T cell (46, 47). Some alternative targeting sites have co-targeting functions that act on tumor cells and tumor microenvironments. For instance, CD123 was expressed in both Hodgkin lymphoma cells and tumor-associated macrophages so that anti-CD123 CAR-T cells could co-target these two kinds of cells and kill them simultaneously (48). The CAR structure is continually evolving to improve the efficacy of current CAR-T cell therapy (32, 49).

## Barriers to Current CAR-T Cell Therapy

Five CAR-T cell products have been approved by the FDA from 2017 to 2021, as listed in **Table 1**. KYMRIAH™ (Tisagenlecleucel) is the first approved CAR-T cell therapy for adult patients with certain types of B-cell lymphoma (50). Three approved CAR-T cell products, YESCARTA™ (Axicabtagene ciloleucel), TECARTUS™ (brexucabtagene autoleucel), and BREYANZI^®^ (lisocabtagene maraleucel), are also approved for the treatment of B cell lymphoma (51–53). The fifth CAR-T cell product, ABECMA^®^ (idecabtagene vicleucel), is used for multiple myeloma therapy (54). Beyond the five approved CAR-T cell products, a large pipeline of CAR-T cells is being studied in clinical trials (55–57), but current CAR-T therapy has several barriers, such as associated toxicity, immunosuppressive tumor microenvironments, and complex manufacturing processes, which hamper the more widespread implementation of CAR-T therapy (58–60).

**Table 1:** An overview of currently approved CAR-T products.

Category	Approval	Target	Indication
Tisagenlecleucel, tisa-cel	Aug. 2017	CD19	B-cell acute lymphoblastic leukemia (ALL) that is refractory or has relapsed after receiving at least second-line regimens; relapsed or refractory large B^-^cell lymphoma (second indication approved in 2018)
Axicabtagene	Oct. 2017	CD19	Treatment in adult patients with relapsed or refractory large B-cell lymphoma (LBCL) Adult patients with relapsed/refractory mantle cell lymphoma (MCL) and B-cell acute lymphoblastic leukemia (ALL)
Ciloleucel, Axi-Cel Brexucabtagene autoleucel, KTE-X19	Jul. 2020	CD19	
Lisocabtagenemaraleticel, L iso-cel	Feb. 2021	CD19	Relapsed/refractory diffuse large B-cell lymphoma (DLBCL)
Idecabtagene Vicleucel, ide-cel	Mar. 2021	BCMA	Patients with relapsed/refractory multiple myeloma who have received four or more previous therapies, including immunomodulators, proteasome inhibitors, and anti-CD38 monoclonal antibodies

The major toxicities associated with current CAR-T therapy include cytokine release syndrome (CRS), immune effector cell-associated neurotoxicity syndrome (ICANS), and on-target/off-tumor toxicity (61–63). CRS is caused by the generation of massive inflammatory cytokines, such as IL-6, IL-10, IL-2, and TNFα, after CAR-T cell treatment. CRS often causes fever, hypotension, hypoxia, organ dysfunction, and even life-threatening adverse reactions (8, 64, 65). The occurrence of severe or life-threatening CRS can reach 25%. ICANS is another common toxicity associated with CAR-T cell therapy and is characterized by neurological abnormalities with aftereffects, usually within 1 week of CAT-cell treatment. The frequent adverse effects caused by ICANS include toxic encephalopathy with aphasia, confusion, and word-finding difficulty (66–68). On-target/off-tumor toxicity is due to the non-special expression of targeting proteins on both normal and malignant cells (69, 70). For instance, when administrating CD19 CAR-T cell in patients with malignant B cells, the on-target/off-tumor effect will lead to B cell aplasia and result in hypogammaglobulinemia due to the eradication of CD19^+^ B cell progenitors by CD19 CAR T cells (71, 72).

The immunosuppressive tumor microenvironment (MVT) inhibits the activation of CAR-T cells and accelerates the exhaustion of T cells (70, 73). Unfavorable factors in immunosuppressive MVT include hypoxia, various immunosuppressive cells, and the sustained expression of co-inhibitory receptors (74, 75). Hypoxia is defined as a shortage of oxygen in the tumor MVT. Immunosuppressive cells in the tumor MVT contain regulatory T cells (Tregs), tumor-associated macrophages (TAMs), and myeloid-derived suppressor cells (MDSCs) (74, 76).

The current manufacturing process of CAR-T cells is a highly complex endeavor, including T cell collection, genetic modification and expansion, and infusion back into patients (77, 78). These multistep technologies and logistics are rife with risks (10). Additionally, the long-term and individualized manufacturing processes pose great challenges for building up standard operating procedures (79). The costly and technology-intensive manufacturing processes of current CAR-T cells make them out of reach for many cancer patients in need of this novel therapy.

## Gene-Editing Tools in CAR-T Cell Therapy

**Figure 2:**
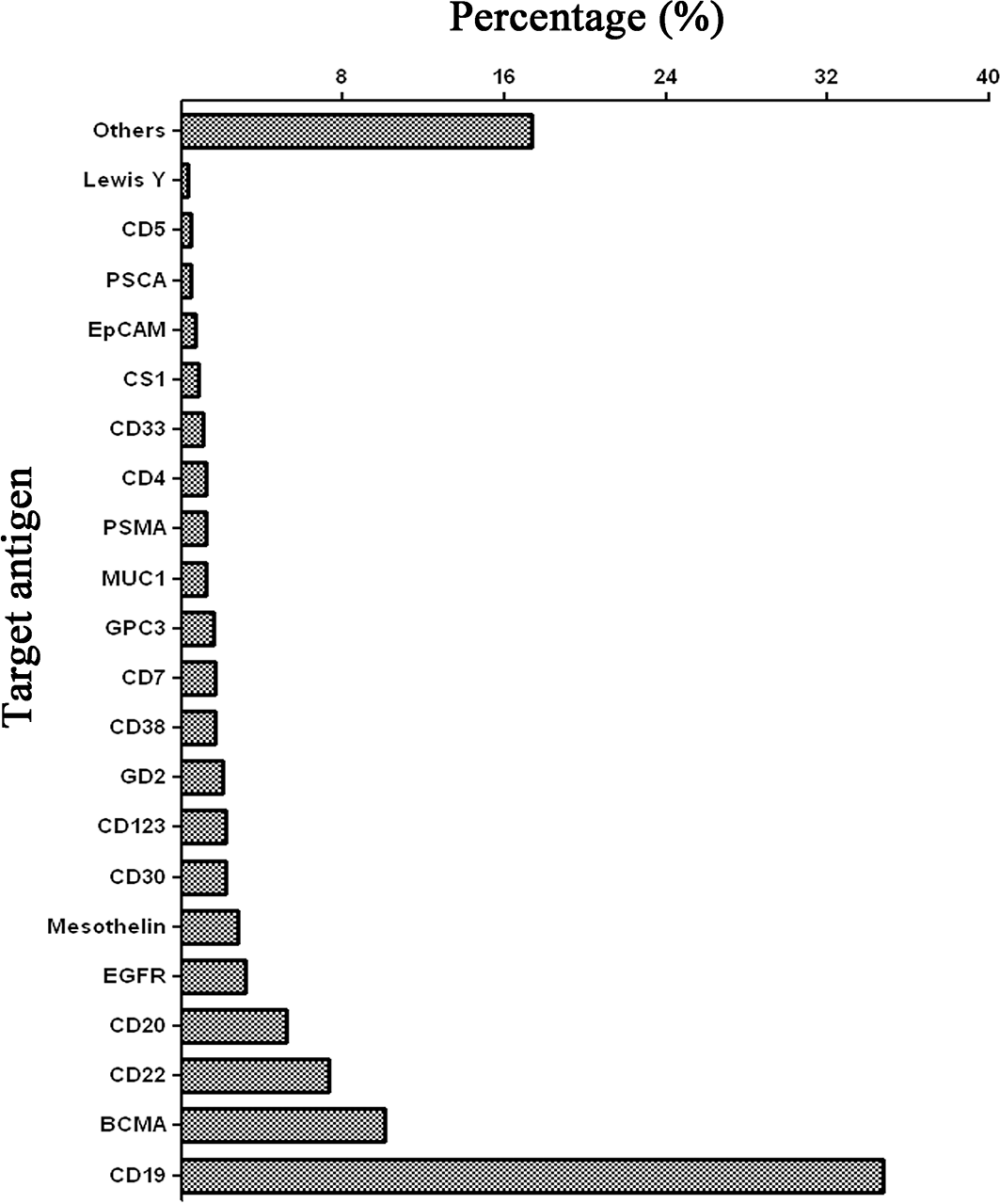
Target antigens of CAR-T cell therapy using CRISPR-Cas9 gene-editing technology registered in ClinicalTrials.gov until June 2021. The data only include clinical trials that are registered in USA.

The gene-editing tools frequently applied to CAR-T cell therapy include zinc-finger nucleases (ZFN), transcription activator-like effector nucleases (TALEN), and clustered regularly interspaced short palindromic repeats-associated 9 (CRISPR-Cas9) technology (80–82). ZFN is the first broadly applied gene-editing tool that includes zinc fingers, a large multimeric protein, wherein each individual finger targets three to four base-pair sequences within genomic DNA (83, 84). Multimeric zinc finger proteins are able to link with the FokI endonuclease to create a ZFN that can cleave site-specific double-stranded DNA and lead to homologous recombination (HR) or non-homologous end-joining (NHEJ) (85). ZFN can achieve effective and specific gene-editing, but it is time-consuming to optimize the targeting protein molecules. TALEN are composed of several TAL units that can recognize base pairs of DNA and link to an endonuclease to generate the site-specific cleavage of DNAs (86, 87). TALEN are more economical than ZFN but still require a long time to optimize the system. CRISPR-Cas9 technology is the most popular gene-editing tool due to its simplicity and efficiency. The CRISPR-Cas9 complex was initially identified as an immune system for cleaving foreign viral DNA in *Streptococcus* pyogenes (88). These CRISPR complexes are first transcribed into RNAs (crRNAs), including bacterial CRISPR sequences, viral sequences (protospacers), and intervening sequences (PAMs) (89). These crRNAs are then complexed with the Cas endonuclease. Once the Cas–crRNA complex recognizes a homologous protospacer and PAM sequence, the Cas endonuclease cleaves the double-stranded DNA, followed by an automatic DNA repair process (88). A short-guide RNA (sgRNA) was introduced into the CRISPR-Cas9 system as the crRNA, making CRISPR-Cas9 an efficient, specific, and simple gene-editing tool (90). The development of gene-editing technology has allowed the precise surgical gene-editing of CAR-T cells to generate exhaustion-resistant T cells *via* removing the PD1 of T cells (91). CRISPR-Cas9 was also used to deplete endogenous antigens, such as CD33 and CD7, in normal cells to reduce the on-target off/tumor toxicity of redirected T cells (92, 93). The CRISPR-Cas9 system has been used in many CAR-T clinical trials involving more than twenty-one target antigens (**Figure 2**) (94–97). CD19 and BCMA account for nearly one-half of the CAR-T clinical trials on these target antigens. To use the CRISPR-Cas9 system more widely to edit CAR-T cells, efficient delivery methods must be developed.

## Gene Delivery Systems

**Figure 3:**
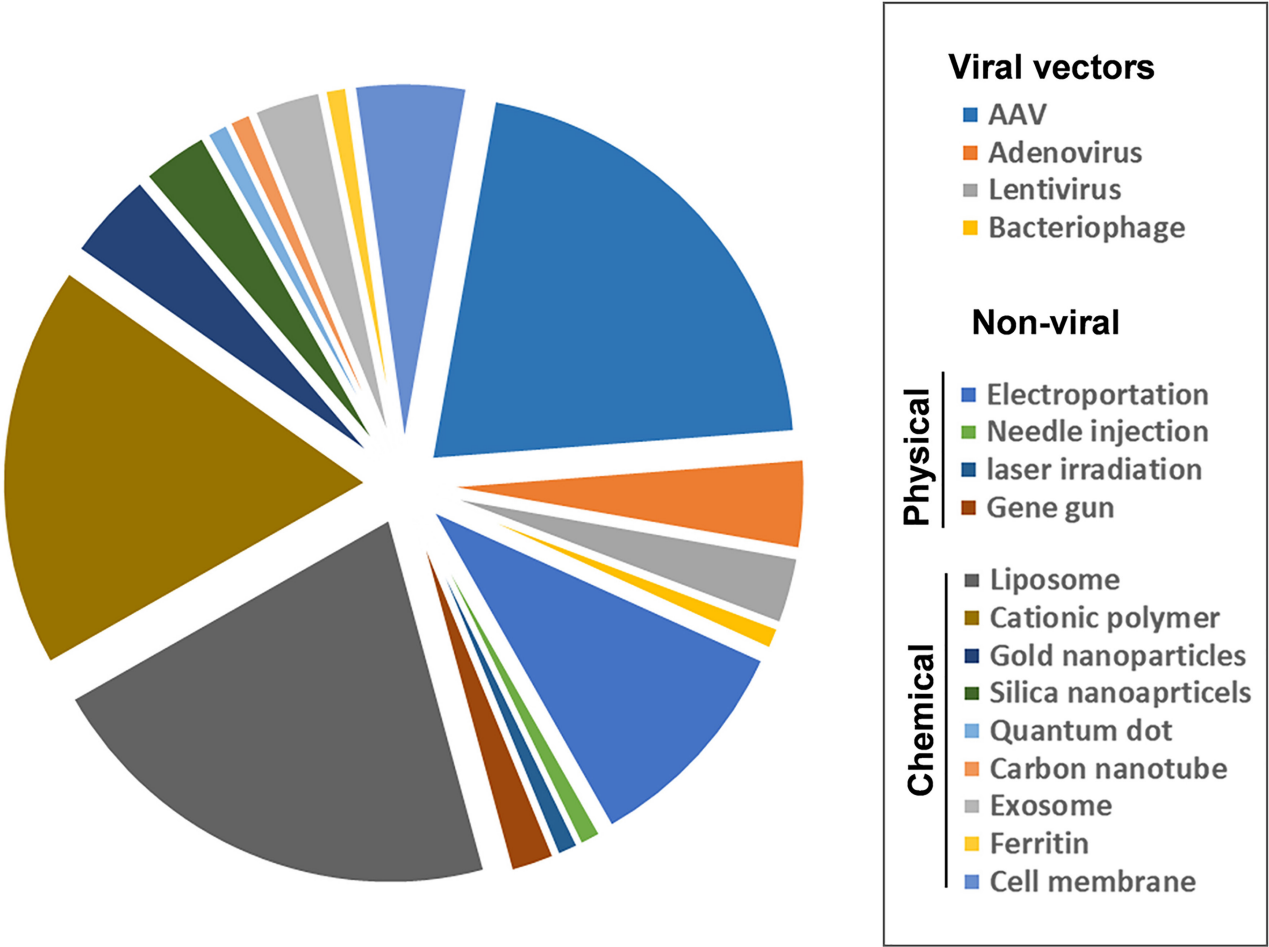
Representation of viral and non-viral nano-delivery systems classified as viral vectors, non-viral (physical and chemical). AAV, adeno-associated virus. The total number of papers is 18,968 obtained from PubMed, and Microsoft Excel was used to obtain the pie graph. The keywords are the name of vectors and gene delivery or CAR gene.

Plenty of delivery systems have been used to deliver gene therapy products including the gene-editing tools and CAR genes (**Figure 3**). Viral vectors have the highest transfection efficiency and have been widely used to deliver genes in various applications (96), but they suffer from the immunogenicity and cellular toxicity. Adenovirus-associated viruses (AVV) have a lower risk of toxicity than other viral vectors such as lentivirus, and adenovirus due to insertional mutagenesis (98). However, the AAV vector has a smaller packaging size (~5.0 kb) than other viral vectors (99). Non-viral delivery systems for gene delivery can be classified into either physical or chemical techniques. Physical techniques include electroporation, needle injection, laser irradiation, and gene guns. Electroporation is one of the most widespread application methods, which induces pore formation on cell membranes and the transient permeability of genes using electric pulses (100–102). Physical techniques have attractive effects on gene delivery due to their low immunogenicity, but they cannot target internal organs. Chemical techniques that mainly use nano-delivery systems include cationic lipids or polymer-based nanoparticles, golden nanoparticles, silica nanoparticles and quantum dots, carbon nanotubes, exosomes, ferritin, and cell membranes. Lipid-based nanoparticles are one of the most attractive non-viral vectors for gene delivery as several formulations of these carriers have been approved to use in the clinic (103–105). Especially, lipid-based nanoparticles have recently been successfully used to deliver SARS-CoV-2 mRNA vaccines (106). Lipid nanoparticles have also been used to deliver the CRISPR/Cas9 system to achieve *in-vivo* genome editing at clinically relevant levels (107, 108). Polymer-based nanoparticles are another system suitable for gene delivery applications. Positively charged polymers can form stable polyplexes with genes that disrupt cell membranes and enable endosomal escape (109, 110). The limitation of polymer-based nanoparticles is their toxicity and immunogenicity caused by the interaction of their positively charged surfaces with negatively charged cell membranes and proteins in blood circulation (111, 112). Exosomes are naturally secreted extracellular vesicles with nanometer sizes that are being extensively investigated as gene delivery vectors due to their natural biocompatibility and minimal immune clearance (113, 114); however, more efforts are required to overcome the difficulties in production, isolation, and purification (115). Cell membranes derived from platelets and red blood cells are biomimetic vectors used for gene delivery that have natural biocompatibility and targeting, but their transfection efficiencies need to be improved (116–118). Each of the other chemical nano-vectors has unique characteristics that determine their effects on gene delivery. Some have shown potential efficiency for the treatment of many diseases, but optimal delivery systems are still unrealized for clinical use.

## *In-Vivo* CAR-T Cell Induction

The current manufacturing process of CAR-T cells requires dedicated equipment and significant technical expertise and is also labor-intensive and time-consuming. (10, 119, 120). It limits the broader worldwide applications of this technology and drives up the price of CAR-therapy, making it out of reach of many patients (121). To simplify the production process, universal CAR-T cells from allogeneic healthy persons were tested in clinical trials, but, the FDA recently halted all clinical trials on the universal CAR-T cells from Allogene due to safety concerns of allogeneic CAR-T cells. There is an urgent need to develop a safe and simple production process for CAR-T cells. *In-vivo* programming of CAR-T cells by nanoparticles is an elegant and novel approach to simplify and standardize the complex manufacturing process of *ex-vivo* CAR-T cells (122). Additionally, the *in-situ* induction of CAR-T cells effectively reduces the systemic toxicity of CRS and ICANS. Recently, *in-vivo* induced CAR-T cells were accomplished through the nano-delivery of CAR structures or gene-editing tools by the team of Matthias Stephan from the Fred Hutchinson Cancer Research Center (Seattle, USA) (18, 20). They accomplished the stable and transient expression of targeting CAR protein in T cells *via* the infusion of nanoparticles loaded with CAR-DNA and CAR-mRNA, respectively. In these two works, the core of the nano-delivery systems was composed of a cationic polymer, poly(β-amino ester), assembled with a second-generation CAR structure targeted to CD19. The exterior of the nano-delivery system was composed of polyglutamic acid (PGA) conjugated with an anti-CD3 antibody. The polymer nanoparticles carrying CD19-specific CAR genes quickly and specifically edited T-cells *in vivo* and brought about comparable antitumor efficacies to conventional laboratory-manufactured CAR T-cells without inducing systemic toxicity. In addition to the polymer nanoparticles, viral vectors such as lentiviruses and AAV have also been tested for the *in-vivo* generation of CAR-T cells. Christian J. Buchholz and his colleagues first reported that lentiviruses encapsulated with a second-generation anti-CD19 CAR gene induced *in-situ* CAR T cells in immunodeficient NOD-*scid*-IL2Rc^null^ (NSG) mice and showed antitumor activity (123, 124). They also exhibited cytokine release syndrome that is notorious in clinical practice. In their study, CAR-positive NK and NKT cells were unexpectedly detected, which were likely caused by the non-specificity of the lentiviral vector. To overcome the non-specificity of the viral vector, Samuel K Lai et al. developed a bispecific binder to redirect the lentiviral vector to T cells for the *in-vivo* specific engineering of CAR-T cells (125). They observed the antitumor activity from the *in-vivo* CAR-T cells engineered by lentivirus, but a relatively low number of CAR-expressing T cells. They considered this to be proof of a valuable and unverified theory of the superior performance and self-renewal capacity of *in-vivo* CAR-T cells compared with that of *ex-vivo* CAR-T cells. However, the toxicity of the *in-vivo* CAR-T cells engineered by the bispecific binder-redirected lentivirus was not included in this work. Among the viral vectors, AVV has a lower risk of toxicity. Xilin Wu et al. recently reported that AAV encoding a third-generation CAR gene could sufficiently reprogram immune effector cells to generate *in-vivo* CAR T cells (126). In this work, they showed a strong proof of concept of AAV-induced *in-vivo* CAR-T cells, but the authors were concerned about the non-specificity of the AAV carrying the CAR gene. Except for the non-specificity of the viral vector, a universal safety concern of viral vectors is the random insertion of genes in the chromosol. Precise and rapid gene editor tools such as CRISPR have been widely used to generate *ex-vivo* CAR-T cells. There are many studies on *in-vivo* gene-editing using CRISPR, but there are still no reports on the application of CRISPR to generate *in-vivo* CAR-T cells. The future applications of combing gene-editing tools and CAR genes will accelerate the clinical adoption of *in-vivo* CAR-T cells.

**Figure 4:**
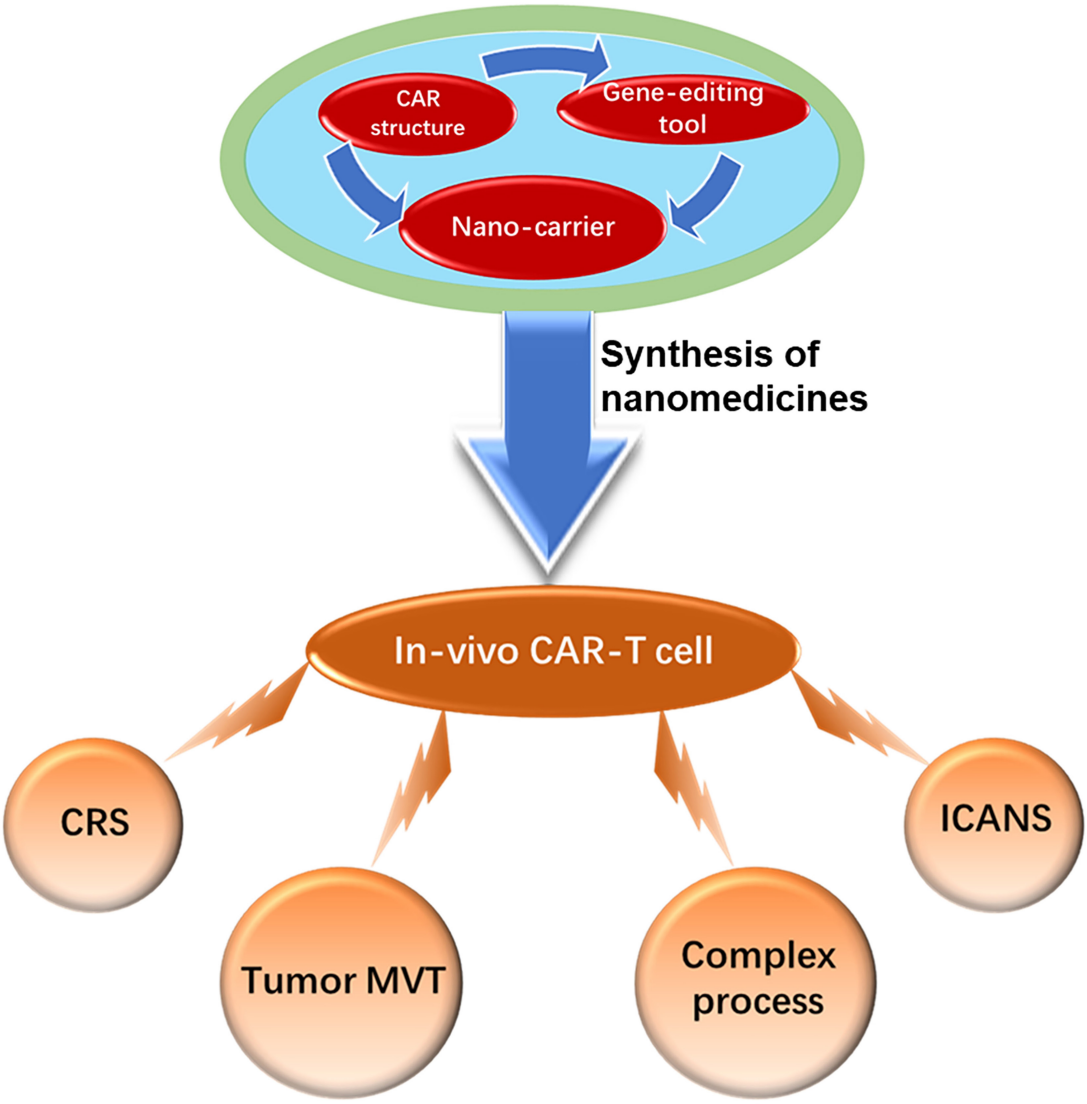
Overcoming the barriers of cytokine release syndrome (CRS), immune effector cell-associated neurotoxicity syndrome (ICANS), tumor microenvironment (MVT), and complex process through *in-vivo* CAR-T cell induced by nanomedicines composed of nano-carrier loaded with the CAR structure or a gene-editing tool.

**Figure 5:**
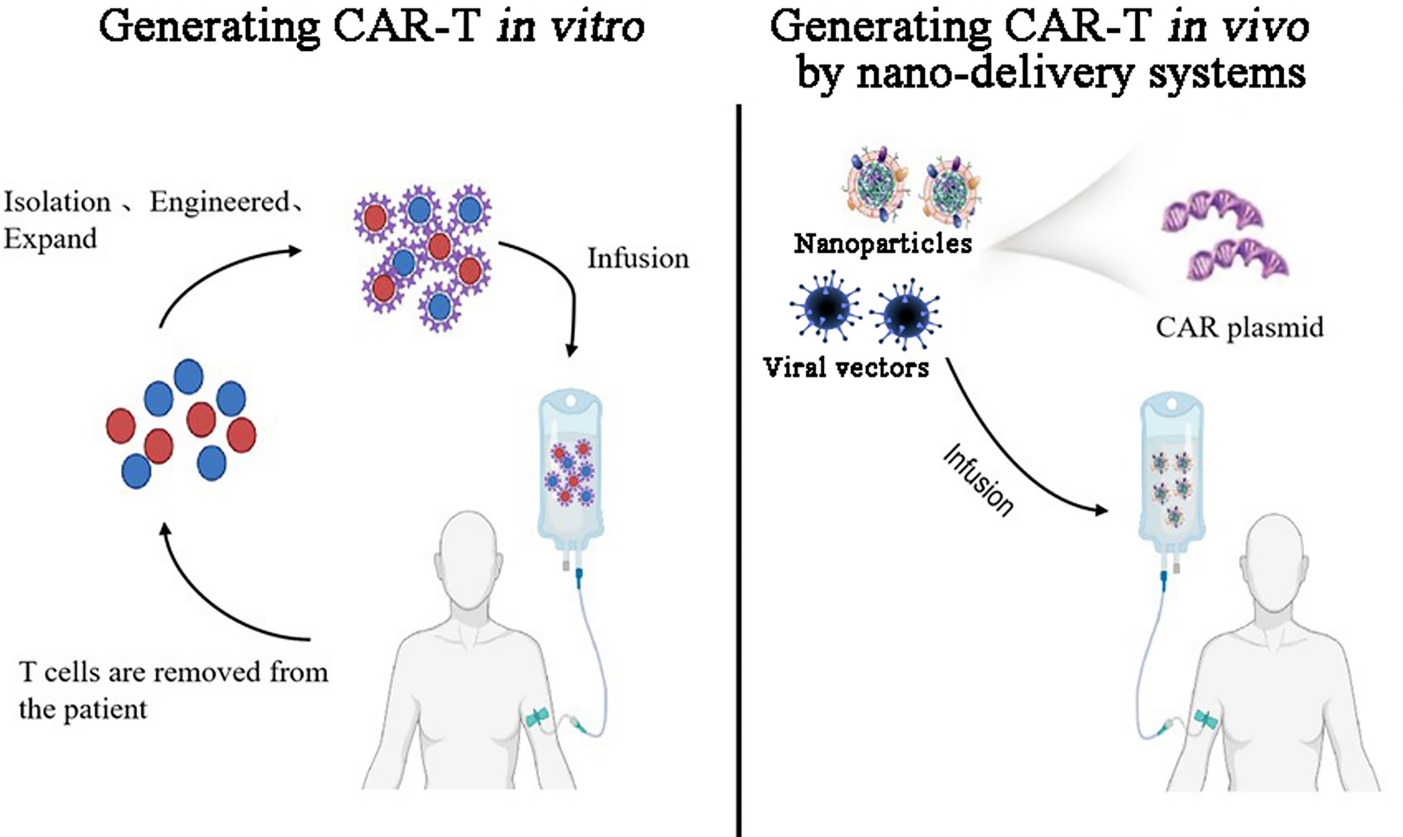
Comparison of generating CAR-T *in-vitro* and generating CAR-T*-in-vivo* by nano-delivery systems: *In-vitro* CAR T cells are first isolated from the patient, proliferated *in-vitro*, and then genetically engineered to screen the successfully edited CAR T cells, which are amplified to a certain number of infusions into the patient. *In-vivo* induced CAR T cells use nanotechnology to encapsulate CAR-expressing plasmids into nano-delivery systems including polymer nanoparticles and viral vectors such as lentivirus and AAV, which are then targeted to tumor regions *in-vivo* to edit T cells *in-situ* at tumor sites to kill tumors.

The nano-delivery of designed CAR-structures and gene-editing tools can induce the *in-vivo* formation of CAR-T cells with multiple functions to overcome the barriers of current CAR-T cells, such as associated CRS and ICANS toxicities, immunosuppressive microenvironment, and complex manufacturing processes (**Figure 4**). Systemic toxicities can be reduced through tumor *in-situ* editing and the expansion of T cells (18, 62). The incorporation of special cytokine genes into a CAR structure enables CAR-T cells to secrete cytokines, flushing the immunosuppressive microenvironment and making it suitable for the survival and proliferation of T cells (127–129). Loading gene-editing tools with CAR structures into nanoparticles can knock out the genes of immune checkpoint blockades to reverse T-cell exhaustion (130–132). More importantly, this approach resolves the difficulty of process standardization and scale-up of the manufacture of *ex-vivo* CAR-T cells (133). The final gene-editor nanoparticles can be conveniently produced, stored, and delivered as usual medicines (**Figure 5**). These studies are just the beginning of the period of *in-vivo* induced CAR-T cells. Their clinical applications still require more efforts to monitor the *in-vivo* editing and expansion status of T-cells.

## Conclusion and Future Prospects

Enormous achievements have been made in CAR-T cell therapy in the last decade, and five CAR-T cell products are available in the clinic. However, current CAR-T cell therapy also has some barriers that need to be overcome such as CRS and ICANS toxicity and expensive and complex manufacturing procedures. The *in-vivo* induced CAR-T cells by nanoparticles loaded with CAR genes and gene-editing tools have shown potential breakthroughs to overcome the abovementioned barriers of current CAR-T cell therapy. Although very few studies have reported nanoparticle-induced *in-vivo* CAR-T cells, robust preclinical data have predicted the future of cellular therapy through nano-delivery approaches. The field of *in-vivo* induced CAR-T cell therapy is still in its infancy with many challenges for the translation of this approach into clinical practice. A systematic summary of the nano-delivery systems for inducing *in-vivo* CAR-T cells can guide the design of the nanoparticles and their cargo to optimize their efficacy (134–136). In summary, *in-vivo* induced CAR-T cells are expected to replace current CAR-T cell therapy and become the standard immune-cell therapy for cancers.

## Author Contributions

TX, LC, CZ, YZ, ZH, and XW performed the discussion. TX and ZH drew the figures. TX, ZH, and XW conceived and wrote the manuscript. All authors contributed to the article and approved the submitted version.

## Funding

This work was supported by the foundation (2019020701011504) from Wuhan Municipal Science and Technology Bureau in China.

## Conflict of Interest

The authors declare that the research was conducted in the absence of any commercial or financial relationships that could be construed as a potential conflict of interest.

## Publisher’s Note

All claims expressed in this article are solely those of the authors and do not necessarily represent those of their affiliated organizations, or those of the publisher, the editors and the reviewers. Any product that may be evaluated in this article, or claim that may be made by its manufacturer, is not guaranteed or endorsed by the publisher.
